# What Do Olympic Shooters Think about Physical Training Factors and Their Performance?

**DOI:** 10.3390/ijerph16234629

**Published:** 2019-11-21

**Authors:** Daniel Mon-López, Francisco Moreira da Silva, Santiago Calero Morales, Olga López-Torres, Jorge Lorenzo Calvo

**Affiliations:** 1 Sport Department, Facultad de Ciencias de la Actividad Física y del Deporte-INEF, Universidad Politécnica de Madrid, 28040 Madrid, Spain; jorge.lorenzo@upm.es; 2 Sport Department, Instituto Superior de Ciências Educativas (ISCE), 2620-379 Ramada, Portugal; franciscosilva36@gmail.com; 3 Postgraduate Management, Facultad de Cultura Física, Universidad Central del Ecuador, Quito 171103, Ecuador; 4 ImFINE Research Group, Health and Human Performance Department, Facultad de Ciencias de la Actividad Física y del Deporte-INEF, Universidad Politécnica de Madrid, 28040 Madrid, Spain; olga.lopez@upm.es

**Keywords:** pistol, qualitative, elite, sport management, fitness coach, shooting, recovery, testing, motor abilities, psychological state

## Abstract

Background: Many aspects affect precision sports like shooting. Skills such as strength and balance are related to shooting performance and therefore, they should be trained. Thus, planned physical workouts can help to improve Olympic Shooting performance. The main objective of this study was to determine elite shooters’ perspective about fitness trainings. Methods: Eight elite international shooters were interviewed using a semi-structured script validated by an expert shooting committee. Their responses were transcribed and analyzed using qualitative methods. The following categories were obtained: fitness training importance on performance, fitness professional support, precompetitive fitness exercises’ orientation and intensity, main motor abilities, recovery process, fitness evaluation and test and physical training influence on the psychological state. Results: The results suggest that athletes consider physical training as a key factor in their performance. Shooters mainly train strength and endurance exercises, depending on the competitions schedule. However, no consensus exists regarding the professional in charge of fitness trainings, nor the recovery methods to minimize performance losses. In addition, general balance trainings or physical condition tests to evaluate the training progress do not seem to be used. Conclusion: We conclude that there should be greater control of fitness training and recovery processes in Olympic shooting.

## 1. Introduction

Olympic shooting demands a high level of precision, constancy and stability from the shooter [1]. In fact, the precision levels are so high that angular errors higher than 0.016° in rifle [2] or 0.066° degrees in pistol [3] can lead to not achieving the score of 10 points. Previous studies have suggested the importance of supporting psychological training [4] and fitness training [5] to improve performance and for shooters to reach their maximum level.

It seems logical to think that the *fitness training importance on performance* could be a critical aspect of shooting [4,6,7]. However, the specific bibliography in this field is limited and results differ according to different studies. Thus, the general aspects related to fitness training (FT) could affect shooting performance by 12% [8]. According to the experts, the importance of FT in shooting performance oscillates between 3% and 46% [9]. Furthermore, the studies which determined muscular influence on shooting performance (maximum score obtained) presented greater discrepancies, with values of 67%–79% when shooters used a 0.22-inch calibre at 50 ft in a pistol standard event [10], 14% in the British Columbia, 48 shot firearms course of fire [11], or 1%–3% when shooters used a 9.0 mm calibre or an air pistol at 10 m [5,7]. 

Regardless of this fact, it is difficult to find specific guidelines about how to train fitness in Olympic shooting. Most of the published literature suggests general guidelines without explaining the intensity or the effects of FT [4]. Other authors have suggested the need to hire *fitness professional support* services to avoid performance losses [12]. The fitness trainer should guide the activities of the shooter obeying the biological, methodological and pedagogical principles to achieve the highest levels of efficiency and performance [12,13]. 

Among the functions of the physical trainer, one would be the control of *precompetitive fitness exercises orientation and intensity* to maximize the shooter´s performance [4,14]. According to the study of Krasilshchikov, Zuraidee and Singh [6], both fitness and technical training should be worked on simultaneously to improve the stability of the pistol [15]. The main FT objectives should be to support the technical training, to overcome competitive stress and to improve the self-image of the shooter [12]. Nevertheless, the literature does not provide clear information about the duration or intensity of the FT. 

Some authors have suggested 20-min sessions of aerobic workouts with heart rates between 110 and 140 beats/minute or strength workouts with low loads and between 15 and 20 repetitions per set [12], while Rio [16] suggested aerobic sessions from 15 to 45 min with heart rates between 120 and 140 beats/minute and strength workouts between 12 and 20 repetitions per set with loads between 60% and 85% of the one maximum repetition (1RM). Regarding *main motor abilities,* strength [5,17], coordination [1], balance [18,19] and endurance [20] seem to be important physical capacities that affect shooting performance. Finger flexors or “hand grip” [21], deltoids abductor force [5], trapezius muscle [22], core and lower body muscles strength [23], seem to be the specific preferential musculature that determines shooting performance.

On the other hand, a *recovery process* is necessary after FT. Resting periods help the body to return to the initial state and to recover the normal levels of homeostasis. However, to the best of our knowledge, no prescription has been described for this sport yet. Active recoveries have been suggested as the best method in clay shooting. Furthermore, other recovery systems such as “stretching”, food supplementation, and resting time or sleeping could be used by the shooters [12]. Nevertheless, the consumption of carbohydrate-electrolyte drinks did not preserve marksmanship accuracy after exercise in other studies [24].

*Fitness evaluation and testing* during the training process is important for the coach to know the level of performance. This information is essential in order to reach the season’s objectives and the progress of the training [25]. Unfortunately, which fitness test are the most accurate is not clear. Studies with a medical-sports character referred to the use of stress tests to determine cardio-circulatory parameters [26]. On the order hand, scientific studies used balance tests [3], the flexor finger strength test [27], the shoulder abductor strength test [5] or even endurance, reaction speed or coordination tests [7] to determine the fitness level of the shooter. Finally, the literature specialized in shooting refers to strength tests (push-ups), joint mobility tests and endurance tests (time to perform 2 km) to evaluate physical condition [12].

Moreover, related to *physical training influence on the psychological state*, it is known that certain psychological aspects influence the performance in shooting (anxiety or self–confidence) [28] as well as some mental states (e.g., mood or wellbeing) [29]. Thus, arousal levels will affect not only the behavior and performance but also the athlete’s wellness [30] and mood [31].

Notwithstanding all the aspects mentioned above, many questions are still unanswered. What are the perceptions of elite shooters in terms of the importance of physical training? Do athletes think that is necessary to hire professional fitness support services? What types of exercises are preferred by the shooters before the competitions? Do the main motor abilities of the literature match the shooters’ opinions? What types of recovery processes are used by the elite shooters? Are the fitness evaluation tests used in shooting sport to control the performance? And lastly, do the shooters think that exercise influences the psychological aspects?

Although previous studies have indicated the importance of physical training and control components in the shooter’s performance, to the best of our knowledge, there is no study that analysed it from the perspective of the elite shooters themselves. Therefore, the aim of this study was to analyze the importance of psychological components from the perspective of the athletes themselves.

## 2. Materials and Methods 

### 2.1. Participants

Eight Olympic shooters (4 men, 4 women) from four different nationalities (Spain 3, Portugal 2, Brazil 2 and Mexico 1) were recruited for the present study. The participants were between 26 and 55 years old (Mode = 34; M = 42 ± 10.54) with a competing experience of (M = 22.75 ± 5.73) years and professional trajectories in first level competitions (Olympic Games, Youth Olympic Games, World Championship and ISSF World Final Cup) (see Table 1). Participants were selected using both social networks and coaches and shooters contacts [32,33]. Invitations were sent to all the shooters informing them about the nature and purpose of the study. Informed consent (attached in Supplementary Materials) was obtained from each participant before the interviews. In order to ensure that athletes were elite shooters with great international experience, two selection criteria were used: (A) have participated at least once in the Olympic Games and (B) meet at least one of the following requirements: (B1) have been selected by their country to participate more than 50 times in top-level competitions and having been a finalist at least one time or (B2) have participated more than 30 times in top-level competitions and having been a medalist once or more times. The competition data of the shooters were obtained from the official web page of the International Sport Shooting Federation [34].

The authors certify that the present study was carried out in the absence of any potential conflict of interest. The study was conducted in accordance with the Declaration of Helsinki and the protocol was approved by the Ethics Committee of the Polytechnical University of Madrid. 

### 2.2. Script of the Interview

The interviews were standardized by using a semi–structured script in order to minimize possible bias. The script questions were designed for both shooters with and without fitness training (Table 2), always from the perspective of the athletes. 

The script included open questions related to several knowledge topics according to the existing scientific literature [19,35]. The selection of the questions was made by consensus [36] of 2 researches who were expert shooting coaches with international experience and subsequently, two external international referees (one expert in qualitative methods and one expert in shooting sport) jointly reviewed the interviews until obtaining the final version of the interview script. Once the final version of the interviews was obtained, it was tested twice in a pilot study to determine the reliability. 

### 2.3. Interviewer and Interview Procedure

A total of 8 semi-structured interviews were conducted by two researchers, who were also Olympic shooting specialists with international experience as coaches, which allowed us to use more specific terminology associated with shooting and generate a relaxed atmosphere with the interviewees. 

A single interview per day was scheduled to avoid possible fatigue. Comfortable and quiet places were selected. One week before the interview, the script was sent to the athletes so that they could respond critically to the last question or suggest new questions. The interviews were conducted by Skype due to distance. Interviews had an average duration of 64 min (the minimum duration was 40 and the maximum 86 min). Interviews were recorded for later transcription with an Olympus WS 852 MP3 recorder. After the transcription and the researcher’s review for recording accuracy, participants were requested to review a copy of their interview transcript [37].

#### Categories

Initial categories were thought to be related to the script interview. Moreover, after interpreting the interviews, the final categories of the study were obtained. Subsequently, from the analysis of the answers related to fitness training, the following categories were obtained: Fitness training importance on performance, fitness professional support, precompetitive fitness exercises orientation and intensity, main motor abilities, recovery process, fitness evaluation and test and physical training influence on the psychological state.

### 2.4. Data Analysis 

This study used qualitative methods that involved an explorative approach to inquiry (Fletcher and Arnold, 2011). The analysis of the data was conducted in four phases: (I) a full transcription of the interviews, (II) a meticulous selection of the phrases and words, (III) elaboration of each category and (IV) a summary of the answers according to each category. An analysis of the transcribed texts was carried out with a Nvivo v.10 software, as well as the frequencies of each idea associated with the study categories. The transcription was made by the same two researchers who made the interviews and revised by a third one as referee. Two researchers analyzed the verbal reports separately and subsequently and both scientists selected the categories from inductive and deductive analysis [38,39]. Cross-triangulation was made by two researchers. In case of discrepancy (only in one category), a third investigator acted as referee. Once the categories were determined, all the concepts, words and phrases were ordered in their respective categories and quantified. 

## 3. Results

The results of the present study were divided into seven different categories related with shooting fitness training.

### 3.1. Fitness Training Importance on Performance

All the athletes referred to the benefits of participating in a fitness program. Even athlete 1, who did not perform any activity, and athlete 5, who rarely practiced, pointed out the importance of good fitness. This concept seems relevant to shooters since the idea of fitness training importance appears on several occasions during the interviews (see Table 3).

I strongly recommend that all people do some physical preparation …

(Athlete 1)

I attribute a lot importance. However, I cannot execute any physical routine in my training…

(Athlete 5)

Great part of the shooters (5/8) had the idea that their physical state would influence performance.

With technical training only, there comes a point where you do not keep going up and cannot improve, then you have to find other methods to improve point by point and one of them is physical and psychological training. It is very important.

(Athlete 2)

Most of the shooters I have met, they do physical and psychological training ... To reach the highest level, you have to complete the technical training with the other two trainings, physical and psychological.

(Athlete 6)

Furthermore, two shooters pointed out that the fitness training was necessary to hold the workouts’ load and avoid injuries, suggesting in some way that endurance training was directly related to the result.

... it is important to achieve some physical endurance to resist the training and competitions and if an athlete does not have a physical preparation, he does not achieve it, or on the other hand he will have the possibility of having an injury ...

(Athlete 3)

Athlete 1 was the exception, declaring himself physically capable of supporting the demands of competition and achieving results without training physical aspects.

### 3.2. Fitness Professional Support

Both the fitness trainer and the physiotherapist were mentioned equally by the shooters when referring to fitness trainings competences (see Table 3). Thus, athletes 3 and 8 were currently working with a fitness trainer in shooting, athlete 4 had a physical trainer during the previous years and nowadays, works in a self-taught way and shooter 6 works with a physiotherapist who is in direct communication with specialists in physical education.

Yes, a physical trainer ... we have this physical trainer since the beginning of this year ... since September ... it was not always like that.

(Athlete 8)

Right now, I am the person in charge, I am the one who do my technical and physical preparation ... About what is physical preparation, I have been studying for many years about it and now I develop my own physical preparation.

(Athlete 4)

On the other hand, shooter 7 did not have a physical trainer. He always had physical training due to his work as air force officer. However, nowadays, he has strength training guided by a gym instructor and the help of a physiotherapist family member. Finally, athletes 1 and 5 acknowledged not practicing any physical activity.

In the military academy I had a guy who was preparing my exercises sets ... thank God he prepared the right series and that did me a lot of good.

(Athlete 7)

### 3.3. Precompetitive Fitness Exercises Orientation and Intensity

Six of the interviewees carried out physical activity oriented towards competition (5/8 regularly). For athlete 3, fitness exercises have two goals: activity oriented towards competition and strength training for injury prevention. According to this shooter, the large amount of technical training allowed him to maintain efficiently the specific endurance. Otherwise, athlete 8 practiced complementary physical activity, but without regularity.

*Not regularly. Since I am shooter, I have not worked constantly due to reasons of time, purely and simply ... Yes, I am training with a physical trainer ...*

(Athlete 8)

Currently I do some exercises for shooting, I do some exercises to avoid injuries that can appear with the shooting practice.

(Athlete 3)

Regarding the intensity of the physical training, most of the interviewees thought that the training had to be well scheduled and had to stop during the moments before the competitions, although they also indicated that the performance was not negatively modified by physical training. However, the exercise intensity was determined by each athlete differently. For example, athlete 4 performed the same type of exercises and decreased the intensity in a previous competition. In contrast, athletes 2 and 6 performed basic training and hired physiotherapy services to recover faster.

It’s just planning well, let’s say that is oriented to the competition. Before a competition, a few days before, 3 or 4 days before, I don’t lift high loads, I work endurance–strength instead of pure strength.

(Athlete 4)

We do not let reach the fatigue to that point in which I cannot feel well ... I can get tired, but the support of the physiotherapist helps me.

(Athlete 6)

### 3.4. Main Motor Abilities

Six shooters highlighted endurance (cardiovascular workouts) and strength as complementary physical capacities in fitness training and necessary to reach high performances (see Table 3).

I combine a lot of aerobic training with strength exercises ...

(Athlete 4)

With the national team and their fitness trainers we have made a great variety of exercises, like gym trainings with different equipment and circuits, bicycle, weights, abs etc. and outdoor we used to do climbing, badminton, swimming, hiking, Pilates and so on ...

(Athlete 2)

I never went too much to the fitness centre, but I always had aerobic activity or something similar.

(Athlete 3)

Regarding the main muscle groups needed in shooting, all shooters refer to the muscles of the arm and forearm, core, abdominals, waist and shoulders.

Muscles of the arm, forearm and shoulder. The latissimus dorsi must be strengthened for the posture too.

(Athlete 1)

### 3.5. Recovery Process

The recovery methods used were different depending on the athlete interviewed. Therefore, we found athletes who did not perform specific recovery actions.

... it’s usually at the hotel, I lie down in bed and I wake up the next day.

(Athlete 1)

At the beginning, after competing I used to end “really tired”, fatigue psychologically and I had to sleep to recover myself…, I didn’t do anything special, only sleep.

(Athlete 2)

On the contrary, other shooters used different recovery methods depending on their necessities. Stretching, cold and hot contrast baths and massages were some of these methods. Moreover, athlete 5 pointed out the need to use techniques such as massage, although for lack of time, he did not perform them.

*No, I stretch before training daily. When I finish the competition, I use other methods … Ice only when I am injured. If I’m not injured, I did not used any recovery method.*

(Athlete 7)

*I think there should be rest after the competition ... muscle recovery should be accompanied by massage, but I do not have time.*

(Athlete 5)

### 3.6. Fitness Evaluation and Test

The results of the interviews show that athletes do not usually perform a physical condition test (3/8). This idea was reinforced by the number of times that athletes mentioned this concept (see Table 3).

Unhappily I say it, because I would like to test ourselves more often … Moreover, I have not been tested physically for a long time.

(Athlete 6)

Even I’m scared, no, I made them once in the CAR (high performance center) ... I remember I did a bicycle and some strength exercises too, and I am not sure, but I remember that it was for a study, in any case it was a long time ago.

(Athlete 5)

At the beginning of the year ... both medical and physical evaluation.

(Athlete 2)

The one who made the fitness tests was the physical trainer and he must do the evaluation again.

(Athlete 8)

### 3.7. Physical Training Influence On the Psychological State

All the athletes agreed that the lack of a physical condition had an influence on the psychological state or mental wellbeing of the shooter, although not all shooters experienced it in the same way. For example, athlete 3 indicated that this relationship was very personal to each shooter, their experiences and their needs to perform physical activity.

I think it is very individual, if the person believes that physical training is important, it will be important. If the athlete believes that this is not important, that will not disturb him.

(Athlete 3)

On the other hand, other athletes referred to physical training’s influence on the wellbeing of the person, but they highlighted the importance of psychological aspects above the physical aspects in competition.

I do not see much importance ... when you feel physically well, you have the tendency to feel wellness, to feel better with yourself. In that sense only it is important. The day of the competition I have never felt much difference by physical training. The psychological aspects affected me more than the physical ones.

(Athlete 7)

Even shooters who did not perform physical activity pointed out the existence of this relationship between the physical and psychological aspects.

I never used to do sports… but I live with a person who has the habit of doing sports and I know that, if he is not running every day, nobody can stand him, so it has a direct relationship with the wellness… So, if I made more fitness it would be better ... the physical training makes you feel more confident and feel better in general.

(Athlete 5)

## 4. Discussion

Most of the shooters (5/8) think that the FT is related to performance and have work-oriented FT before competition, which agrees with the previous literature [4,6,7]. They also think that the quantification of the FT is important to performance, reporting sentences such as, “It is very important” or “I attribute a lot importance”. Nevertheless, there is no consensus in the literature when it comes to quantifying it. Some studies suggested great percentages [9], while others confered small percentages of influence [8,9]. Shooters also think that physical condition has an influence on psychological state and mental wellbeing. Despite the importance given, there is no consensus about the professional support of physical training and some shooters do not have a professional specialist in charge when they are training. Other times, it may not be someone from the specific area (e.g., physiotherapist). Nevertheless, the bibliography recommends hiring specific fitness professional support [12] and therefore, federations and shooting organizations should have this aspect in consideration to improve their athlete’s performance.

Precompetitive fitness exercises orientation and intensity should be oriented and under control to avoid planning errors and performance loses [4]. In our study, 6/8 shooters carried out physical activity oriented towards competition, but only 5/6 work regularly, combining technical training with physical training, as previous studies have suggested [6]. Working simultaneously fitness and technical aspect improves the stability of the pistol [6] and consequently, shooting performance [15]. It is important to remember that minimal performance differences in precision sports can mean success or failure [40]. Despite differences in the intensity used for the shooters, all of them pointed out that performance was not negatively modified by physical training. 

The main motor abilities trained, according to the shooters, were aerobic capacity and strength. The literature in this regard is scarce. Some authors recommended aerobic trainings [20] with low to medium heart rates [12,16], isometric strength [5,17] and strength workouts with low loads [12] or medium loads [16]. Nevertheless, although Olympic shooting is a sport that requires balance [18,19] and coordination [1], athletes mentioned these abilities less than the other qualities mentioned above. This could be due to the lack of specific work in this area (not all the shooters hired professional fitness support) or because shooters perceive strength and endurance as more important shooting skills.

On the other hand, the importance of the relationship between the musculature and the shooting performance varies according to different authors from high [10], to medium [11], to small [5,7]. The shooters in our study mentioned that the most needed muscle groups in shooting were the arm, forearm, core, abdominals, waist and shoulders. These results are in accordance with previous studies which highlighted finger flexors or “hand grip” strength [21], deltoids abductor force [5], trapezius muscle activation [22] and the core and lower body muscles [23].

As in other sports, Olympic shooters need a recovery process after trainings or competitions. Kilty [12] has suggested food and beverages supplementation and the use of rest time or sleep. According to our data, shooters used very few recovery techniques. Stretching, cold and hot contrast baths and massages are some applied methods. Moreover, sometimes they do not use any recovery method due to lack of time. Therefore, it seems that elite shooters could consider introducing other methods with scientific evidence in other sports to improve their recovery and performance [41,42]. 

Fitness evaluation and tests are common in many sports in order to control the training load and athlete’s physical state. However, according to our data, only 3/8 shooters perform a physical condition test. In fact, shooters who did a fitness test pointed out that this test were carried out by the physical trainer or by professionals with high performance facilities. These results are contrary to the previous studies that suggested the use of general tests [12,26], specific balance test [3], strength test [5,27] or general tests such as the 20 m shuttle run test (20 MST), the alternate hand wall toss test or reaction speed tests [7] to determine the fitness level of the shooter. Thus, shooters could consider introducing some methodological resources to improve trainings and performance control.

Another important aspect is the influence of physical training on the psychological state. All the shooters in our study agreed that psychological state and mental wellness could be modified by physical activity. This influence depended on each individual according to their experiences. The three main aspects referred by the shooters were self-confidence, feeling wellness and feeling better in general and with yourself. Similarly, Kilty [12], stated that FT could improve the sel-image of the shooter. In the same line, Feltz [29] indicated that the effect of some mental states (e.g., mood or wellbeing) could indirectly influence sport performance. Furthermore, these mental states of wellness [30] and mood [31] are modifiable by physical trainings. 

Although our participants are very high-level athletes, some limitations should be mentioned about our study. Even though the interviews were sufficiently extensive, more questions could have been asked in some categories. Moreover, it is difficult to make assumptions on what type of training shooters should complete, as we only interviewed eight elite shooters and maybe we did not have enough details about the activity they completed. Furthermore, only precision shooters were analyzed in this study. Hence, it would be interesting to study other shooting modalities in the future.

## 5. Conclusions

In general, shooters agree that fitness training is an important factor related to shooting performance and mental state. Normally, shooters have aerobic and strength work-oriented fitness trainings, mainly for arms and abdominal muscles, but not general balance trainings. However, athletes do not usually perform a physical condition test, nor do they use recovery techniques in a planned way. Moreover, not all the shooters hired support services and there is no consensus about the professional in charge of physical trainings. Thus, to improve elite shooter performance, federations and shooting responsible nations or clubs should consider greater control over fitness trainings and recovery process in Olympic shooting.

## Figures and Tables

**Table 1:** Participants profiles, number of participations in first level international competitions, times finalist and medals.

Athlete	Sex	Age (y) (X = 42 ± SD = 10.54)	Start of Competing Age (y) (X = 22.8 ± SD = 5.73)	JJOO/YOG	WCH	EUCH/AMCH/PAN Games/EU Games	WC	FWC
1	M	54	26	5P 2F –	6P 3F 1S	35P 27F 3G, 2S, 8B	52P 29F 4G, 5S, 5B	7P 8F 2S
2	F	56	27	5P 1F –	4P – –	22P 3F –	52P 27F 1G, 4S, 4B	5P 5F 1B
3	M	26	14	1P, 1P(YOG) 1F, 1F(YOG) 1S, 1S(YOG)	3P 1F –	4P 2F 1G, 1S	29P 3F 2G	1P – –
4	M	40	24	2P – –	5P 2F 1G	13P 6F 1G, 1S, 1B	58P 8F 1B	3P 3F 1B
5	F	34	16	1P – –	4P – –	23P 1F –	25P 5F –	– – –
6	F	34	19	2P 1F –	4P – –	8P 7F 3S	27P 7F 1G, 1S, 3B	5P 4F 2G
7	M	49	30	2P – –	5P 3F 1G,1S,1B	9P 22F 7G, 7S, 3B	39P 5F –	– – –
8	F	43	26	1P – –	7P – –	25P – –	19P 1F –	– – –

Note: Abbreviations. M = male, F = female, JJOO = Olympic Games, YOG = Youth Olympic Games, WCH = World Championship, EUCH = European Championship, AMCH = American Championship, PAN Games = Pan–American Games, EU Games = European Games; WC = World Cup, FWC = Final World Cup; P = number of participations in the competition, F = times being finalist in the competition. G = gold medals won, S = silver medals won, B = bronze medals won, X = arithmetic mean, SD = standard deviation.

**Table 2:** Semi-structured interview script.

**Greetings and thanks…**
*Specific questions of the interview in the psychological section*
What is your opinion about the fitness training in Olympic shooting? Do you think that the fitness training can affect the performance?
**End of the interview**
There is some question that was not asked, and you wish it had been made?

**Table 3:** Concepts and words most repeated by the athletes interviewed in the fitness area.

Category	Ideas/Concepts/Words	Sh 1	Sh 2	Sh 3	Sh 4	Sh 5	Sh 6	Sh 7	Sh 8	Total
FTIOP	Technical training	11	11	13	9		3	21	1	69
	Technical training as fitness training	11	15	10	9	6		2		53
	Fitness training importance	5	8	13	7	3	7	5	10	58
FPS	Fitness coach		3	2	4		3	2	4	18
	Phisiotherapist/phisiotherapy			2		3	12	4		21
PFEDI	Training intensity and duration		1	1	1			1	2	6
MMA	Cardiovascular/Aerobic/Endurance		3	13	12		8	10	16	62
	Strength/force/weight training	3	3	1	10	1	7	30	6	61
	Streching/flexibility			2			11	1	1	15
	Balance/equilibrium		6	3	1		7		7	24
RP	Fitness recovery after training–competition	2	5	2	5		3	1	2	20
FET	Fitness control/testing		2		2	1			4	9
PTIPS	Confident	0	6	2	12	6	0	6	0	32
	Nervous	6	5	0	12	2	0	7	1	33

Note: Abbreviations. Sh = shooter. Fitness training importance on performance (FTIOP), Fitness professional support (FPS), Precompetitive fitness exercises orientation and intensity (PFEDI), Main motor abilities (MMA), Recovery process (RP), Fitness evaluation and test (FET) and Physical training influence on the psychological state (PTIPS).

## Supplementary Materials

The following are available online at https://www.mdpi.com/1660-4601/16/23/4629/s1, Written consent S1: template permission form.
