# Supplementary material for: What Do Olympic Shooters Think about Physical Training Factors and Their Performance?
Source: Int J Environ Res Public Health. 2019 Nov 21;16(23):4629. doi: 10.3390/ijerph16234629 (PMC6926526; doi:10.3390/ijerph16234629)
Supplement: Supplementary file 1 [file ijerph-16-04629-s001.pdf]

UNIVERSIDAD POLITÉCNICA DE MADRID

FACULTAD DE CIENCIAS DE LA ACTIVIDAD FÍSICA Y DEL DEPORTE

DEPARTAMENTO DE DEPORTES

Madrid, 17 de octubre de 2019

Estimado/a:

Me dirijo a ti en primer lugar dándote las gracias por emplear tu tiempo en la lectura de este documento. Por medio del mismo, te informaremos de una investigación que estamos llevando a cabo desde la Universidad Politécnica de Madrid.

Ruego leas atentamente la siguiente documentación, la hoja de información al participante y el consentimiento informado, y optes por participar para ayudarnos a avanzar en el conocimiento de nuestro deporte.

Recibe un cordial y atento saludo.

El Investigador Principal:
Daniel Mon-López

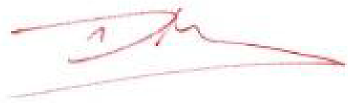

HOJA DE INFORMACIÓN AL PARTICIPANTE

PLAN DE INVESTIGACIÓN

El objetivo de este proyecto será analizar distintos factores que influyen en el rendimiento deportivo en tiro olímpico.

¿QUIÉNES SOMOS?

A continuación, se detallan algunos datos profesionales y académicos del investigador principal a modo de presentación:

Investigador: Daniel Mon López.
Categoría: Profesor Ayudante Doctor en la UPM.
Departamento: Deportes.
Facultad: Ciencias de la Actividad Física y del Deporte**.**
Docencia: El objetivo de este proyecto será analizar distintos factores que influyen en el rendimiento deportivo en tiro olímpico.
Perfil investigador: Análisis del rendimiento aplicado al deporte.

¿QUÉ BUSCAMOS?

Este documento tiene la finalidad de ofrecerte información sobre un estudio de investigación, en el que se te invita a participar: "Importancia del entrenamiento físico en el tiro deportivo desde la perspectiva del atleta". Investigación que ha sido aprobada por su Comité de Ética de la Investigación.

Si decides participar en el mismo, tienes derecho a recibir información personalizada del investigador, **LEER ANTES ESTA HOJA DE INFORMACIÓN AL PARTICIPANTE** y hacer todas las preguntas que estimes oportunas para comprender el mismo. Si así lo deseas, puedes llevarte el documento, consultarlo con otras personas, y tomar el tiempo necesario para decidirte sobre tu participación. La participación en esta investigación es completamente **VOLUNTARIA**.

¿CUÁL ES EL OBJETO DEL ESTUDIO?

En la importancia dada al entrenamiento físico, existe una gran congruencia atribuida a sus efectos positivos en los resultados, principalmente en la prevención de lesiones, su repercusión en el estado psicológico y los beneficios para la salud. Sin embargo, la percepción en el mundo del tiro es que no todos los deportistas lo practican y no todos entrenan acompañados por especialistas en ejercicio físico. Es importante conocer desde la perspectiva del atleta que beneficios proporciona el entrenamiento físico y los principales grupos musculares que deben hacer ejercicio, así como que tipos de evaluación de la condición física utilizan la mayoría de los tiradores.

¿CUÁLES SON LOS OBJETIVOS DE LA INVESTIGACIÓN?

El presente artículo de investigación tiene como objetivo aclarar la importancia que los atletas olímpicos atribuyen al entrenamiento físico para lograr un excelente rendimiento en sus competiciones.

¿CÓMO SE PLANTEA EL ESTUDIO?

El diseño de la investigación responde a un método mixto en el que se combinarán un enfoque cualitativo y cuantitativo a través de diferentes fases:

Fase I. Entrevistas personales: cuyo propósito es indagar sobre los agentes del entrenamiento físico y psicológico percibidos por los deportistas de tiro como factores de rendimiento. A través del análisis de sus aportaciones, identificaremos también los recursos y estrategias con los que cuentan para afrontar las competiciones.

Fase II. Transcripción y análisis: Posteriormente se realizará una transcripción y análisis por categorías de los conceptos *expresados por los atletas en relación a la preparación física y psicológica que determinan el rendimiento desde la perspectiva de los deportistas.*

Fase III. Elaboración de conclusiones: Finalmente con todos los resultados se elaborarán resúmenes y conclusiones acerca de las opiniones de los deportistas entrevistados.

¿POR QUÉ SE TE OFRECE PARTICIPAR?

Te hemos invitado a participar por ser **UN INFORMANTE CLAVE PARA LA INVESTIGACIÓN**. El estudio se centra en los deportistas que ha competido internacionalmente al más alto nivel o que han disputado JJOO durante su trayectoria deportiva. Por sus conocimientos y trayectoria profesional, su colaboración y participación es imprescindible para alcanzar los propósitos de la investigación descritos anteriormente.

¿EN QUÉ CONSISTE SU COLABORACIÓN EN EL ESTUDIO?

Requerimos de su consentimiento para participar en este estudio, siendo entrevistado para que, desde su visión personal y experiencia laboral, aborde la temática en relación al objeto de estudio informado.

Esta participación se materializa en una **ENTREVISTA PERSONAL,** que tendrá una duración en torno a 60 minutos. La entrevista será **GRABADA EN AUDIO**, siempre y cuando se acceda a dicha situación, al inicio de la misma se explicará la metodología de trabajo. Una vez transcrita se enviará una **COPIA DE LA ENTREVISTA,** por si se estima oportuno, realizar alguna matización de lo expresado por parte del participante.

El **INFORME** que resulte de la entrevista, será tratado con absoluta **RESERVA y CONFIDENCIALIDAD**. Se usará un código para identificar a cada uno de los entrevistados con el objeto de cubrir el **ANONIMATO**. En todo momento la identificación real de los participantes y de sus respectivos puestos de trabajo, estará

custodiada y salvaguardada por los investigadores del estudio (*Ley Orgánica 15/1999, de 13 de diciembre, de protección de datos de carácter personal*).

Asimismo, puede negarse a participar o retirarse de la investigación cuando lo estime oportuno sin expresión de causa. Momento en el que los datos aportados por el participante serán borrados y no utilizados en el estudio.

¿QUÉ OCURRIRÁ CON LOS RESULTADOS DEL ESTUDIO?

Los resultados de la investigación serán presentados y publicados por una vía de producción bibliográfica:

1. Artículos científico-académicos.

¿OBTENDRÉ ALGÚN BENEFICIO POR PARTICIPAR?

A todos los participantes del estudio se les entregará una vez finalizada la investigación, el informe final de la misma en formato digital.

¿QUIÉN ME PUEDE PROPORCIONAR MÁS INFORMACIÓN?

Para cualquier duda o aclaración, puedes contactar con el investigador Daniel Mon López a través del:

Teléfono de contacto: 91 497 7597

E-mails: daniel.mon@upm.es

Contacto del comité de Ética a todos los participantes (secretaria.adjunto.vinvestigacion@upm.es).

CONSENTIMIENTO INFORMADO

DATOS DEL ESTUDIO PARA EL QUE SE OTORGA EL CONSENTIMIENTO:

Investigador Principal: Dr. Daniel Mon López.

Plan de Investigación: Importancia del entrenamiento físico en el tiro deportivo desde la perspectiva del atleta.

DATOS DEL PARTICIPANTE:

Nombre Participante

____

D.N.I.

____

1. Declaro que he leído la Hoja de Información al Participante (HIP) sobre el estudio citado y acepto participar en él.
2. Se me ha entregado una copia de la Hoja de Información al Participante (HIP) y una copia de este Consentimiento Informado (CI), fechado y firmado. Se me han explicado las características y el objetivo del estudio.
3. Se me ha dado tiempo y oportunidad para realizar preguntas. Todas las preguntas fueron respondidas a mi entera satisfacción.
4. Sé que se mantendrá la confidencialidad de mis datos y que mi participación es anónima.
5. El consentimiento lo otorgo de manera voluntaria y sé que soy libre de retirarme del estudio en cualquier momento del mismo, por cualquier razón. En tal caso, los datos aportados por el participante serán borrados y no utilizados en el estudio.

DOY □

NO DOY □

Mi consentimiento para la participación en el estudio propuesto.

Madrid, 17 de octubre de 2019

Firma del participante:
